# Holocene forest dynamics in central and western Mediterranean: periodicity, spatio-temporal patterns and climate influence

**DOI:** 10.1038/s41598-018-27056-2

**Published:** 2018-06-12

**Authors:** Federico Di Rita, William J. Fletcher, Josu Aranbarri, Giulia Margaritelli, Fabrizio Lirer, Donatella Magri

**Affiliations:** 1 Department of Environmental Biology, Sapienza University of Rome, Piazzale Aldo Moro, 5, 00185 Roma, Italy; 2 Department of Geography, School of Environment, Education and Development, The University of Manchester, Oxford Road, Manchester, M13 9PL UK; 3 Department of Geography, Prehistory and Archaeology, University of the Basque Country, C/Tomás y Valiente s/n, 01006 Vitoria-Gasteiz, Spain; 4 Istituto per l’Ambiente Marino Costiero (IAMC), Consiglio Nazionale delle Ricerche, Calata Porta di Massa, Interno Porto di Napoli, 80133 Napoli, Italy

## Abstract

It is well-known that the Holocene exhibits a millennial-scale climate variability. However, its periodicity, spatio-temporal patterns and underlying processes are not fully deciphered yet. Here we focus on the central and western Mediterranean. We show that recurrent forest declines from the Gulf of Gaeta (central Tyrrhenian Sea) reveal a 1860-yr periodicity, consistent with a ca. 1800-yr climate fluctuation induced by large-scale changes in climate modes, linked to solar activity and/or AMOC intensity. We show that recurrent forest declines and dry events are also recorded in several pollen and palaeohydrological proxy-records in the south-central Mediterranean. We found coeval events also in several palaeohydrological records from the south-western Mediterranean, which however show generally wet climate conditions, indicating a spatio-temporal hydrological pattern opposite to the south-central Mediterranean and suggesting that different expressions of climate modes occurred in the two regions at the same time. We propose that these opposite hydroclimate regimes point to a complex interplay of the prevailing or predominant phases of NAO-like circulation, East Atlantic pattern, and extension and location of the North African anticyclone. At a larger geographical scale, displacements of the ITCZ, modulated by solar activity and/or AMOC intensity, may have also indirectly influenced the observed pattern.

## Introduction

Understanding the long-term trends and spatial patterns of climate variability in the current interglacial period, the Holocene, is crucial to assess the significance of ongoing climate change and future projections. Furthermore, taking into account the commonality of processes and mechanisms shared by climate models at all timescales, understanding the low frequency (millennial) component of past climate change is ultimately essential for improved predictions on all timescales. While it is well-known that the Holocene exhibits a millennial-scale climate variability, its periodicity, spatio-temporal patterns and underlying processes are not fully deciphered yet.

- Periodicity: since the pioneering studies of Bond *et al*.^1^ in the North Atlantic region, the nature of climate variability was associated to a quasi-periodic 1500-year cycle (1470 ± 500, Bond cycle), which was tentatively attributed to solar activity, ocean current intensity variations, tidal forcing, atmospheric processes linked to the North Atlantic Oscillation (NAO), or modifications of the geomagnetic field^1–3^. In Europe and in the Mediterranean regions, the Bond cycles have been often associated to millennial scale warm/cold and humid/arid climate shifts documented in many marine and continental records^4–6^. According to Magny *et al*.^5^ these recurrent climatic events coincided with decreases in solar activity and deglacial outbursts in the North Atlantic area during the interval 11,700–7000 cal BP, and to a possible combination of NAO-type circulation and solar forcing from ca. 7000 cal BP onwards. Debret *et al*.^7^, detected a new pervasive low-frequency millennial scale oscillation of 1600–1800 yr after 6 ka in several records from the North Atlantic region.
- Spatial pattern: the spatial pattern of the impact of millennial-scale climate cycles over the Mediterranean Basin is another intriguing issue. Recent studies have shown climate oscillations that were not always clearly recorded across the entire Mediterranean Basin or exhibited opposite trends in different regions^8,9^. Magny *et al*.^5^ reveal North-South palaeohydrological contrasts in the central Mediterranean during the Holocene and suggest a latitudinal divide at ca. 40°N. They also suggest possible latitudinal shifts in the limit between the contrasting hydrological sectors of the Mediterranean in response to changing NAO. Fletcher *et al*.^9^ highlight contrasting hydrological signals between the western and south-eastern sectors of the Mediterranean and suggest that prevailing or predominant phases of NAO-like circulation conditioned the climate pattern of the Mediterranean after 6000 cal BP by modulating the long-term trend of intensity and position of the westerlies. Di Rita and Magri^10^, reviewing the patterns of a forest decline event between 4500 and 4000 cal BP in many sites of the central Mediterranean south of 43°N, attribute it to the expansion or northward displacement of a North African high-pressure cell, inducing arid climate. This forest decline corresponds to a transition from positive to negative NAO culminating around 4.2 ka cal BP^11^.
- Climate processes: the recognition of the 4.2 ka event in proxy records from North America, through the Middle East to China, and from Africa, parts of South America to Antarctica^12^ suggests that past environmental changes in the Mediterranean may reflect the influence of climate dynamics at the global scale. Similar considerations were also advanced by Roberts *et al*.^13^, who suggest that the Little Ice Age (LIA)/Medieval Climate Anomaly (MCA) hydroclimatic pattern in the Mediterranean was determined by a combination of different climate modes along with major physical geographical controls, and not by NAO forcing alone. In the south-central Mediterranean, the current hydrological regime is characterized by summer drought and winter precipitation, also influenced by the NAO variability (e.g.^14^), although Trigo *et al*.^15^ argue that the precipitation regime in some sectors of the south-central Mediterranean (e.g., Sicily) cannot be explained by the NAO pattern. However, in south-central Mediterranean a correlation of the precipitation with the NAO index has been documented^16,17^. Brandimarte *et al*.^17^, have found that during the last century, although the Italian Peninsula is generally negatively correlated with NAO, Eastern Sicily and Northern Africa show a positive correlation. López-Moreno *et al*.^16^ have demonstrated an important instability in the influence of positive and negative phases of the NAO on Mediterranean precipitation variability and droughts of the past century, reflecting interdecadal changes in the position of the main pressure centres that characterize the NAO. They also underline the importance of considering other climate teleconnections to explain the precipitation anomalies in different sectors of the Mediterranean region, including East Atlantic pattern (EA), ENSO, and Western Mediterranean Oscillation (WeMO), which are considered among the dominant climate modes currently acting in the Mediterranean^18^.

More detailed palaeoenvironmental and palaeoclimate marine and continental records, as well as modelling studies are needed to test the millennial scale periodicities and explore possible influences of different general atmospheric circulation patterns. In the Mediterranean, palaeovegetation records provide a remarkably sensitive proxy to reconstruct changes in atmospheric conditions linked to the different modes of climate variability, especially those influencing the palaeohydrological regime and seasonality of precipitation, which induce changes in vegetation composition and structure, as well as in plant distributions.

**Figure 1:**
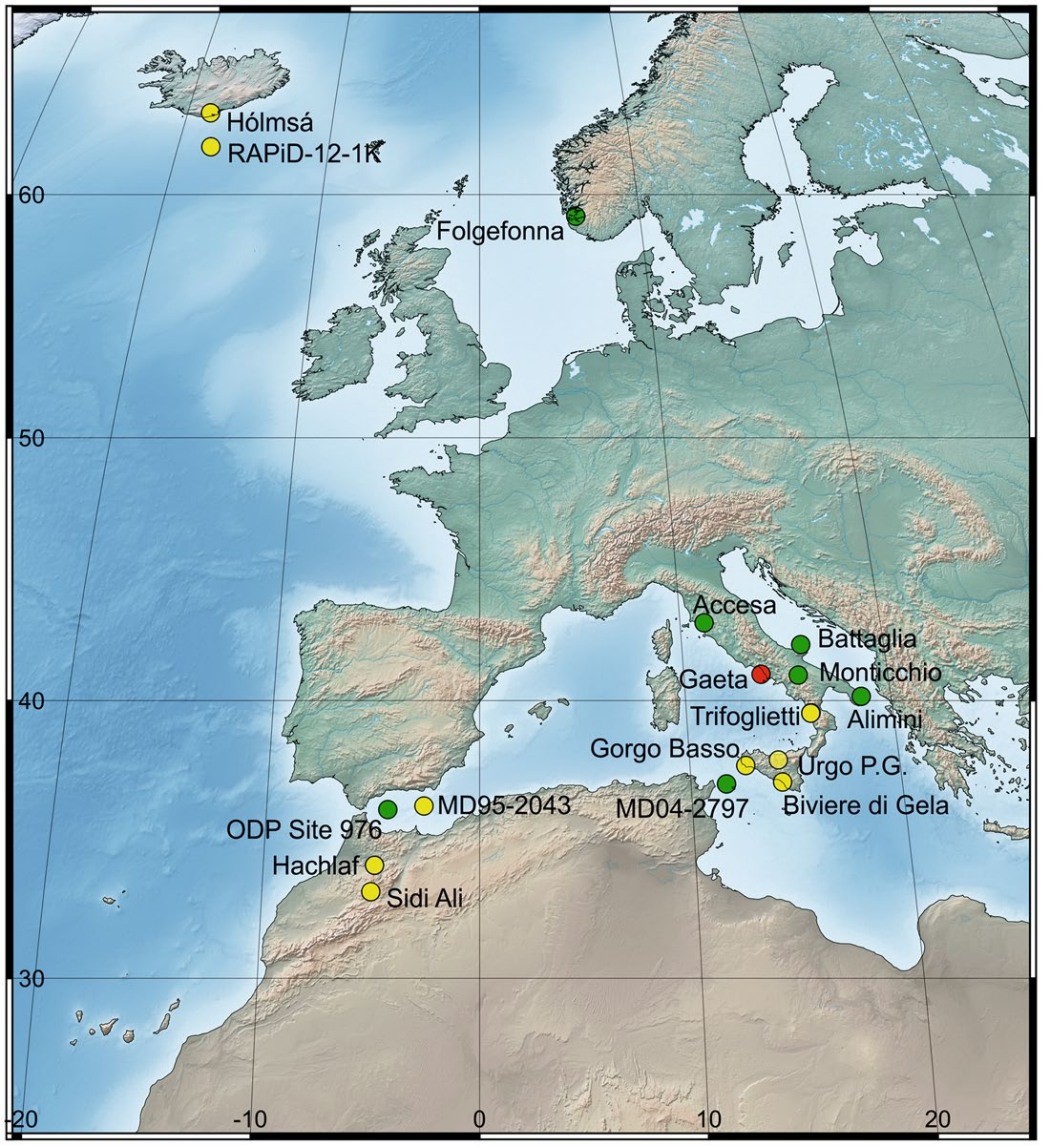
Location of the sites mentioned in the text. Gulf of Gaeta record (red dot), sites shown in Fig. 5 (yellow dots: Sidi Ali^23^; Hachlaf^28^, site MD95-2043^9^, Gorgo Basso^34^, Biviere di Gela^35^, Urgo Pietra Giordano^36^, Trifoglietti^37^, core RAPiD-12-1K^43^, and Hólmsá^41^); and other sites mentioned in the text (green dots: ODP site 976^26^, Folgefonna^42^, site MD04-2797^4^, Lago Alimini Piccolo^10^, Lago Grande di Monticchio^38^, Lago Battaglia^39^ and Lago dell’Accesa^40^). Map produced using Corel Draw Suite X8 (https://www.coreldraw.com). The background map (DEM 30 m; Lambert Azimuthal Equal Area projection) was retrieved from Advanced Spaceborne Thermal Emission and Reflection Radiometer (ASTER) Global Digital Elevation Model (GDEM). ASTER GDEM is a product of NASA and METI and is distributed by NASA LP DAAC (https://lpdaac.usgs.gov/dataset_discovery/aster). The final map was generated using the software QGIS version 2.18.15 ‘Las Palmas’ (QGIS Development Team, 2017. QGIS Geographic Information System. Open Source Geospatial Foundation Project https://www.qgis.org/it/site/).

In this study, we discuss the periodicity, spatio-temporal patterns and possible climate mechanisms influencing the forest development in the south-central Mediterranean, based on a 5500-year long palaeovegetational record from a marine core drilled in the Tyrrhenian Sea, compared with other records from the central and western Mediterranean (Fig. 1).

The climate of the Gaeta area is typically Mediterranean with a high spatial variability of weather conditions also due to local cyclogenesis. The most intense, deepest, and most persistent cyclones are observed in wintertime, frequently associated with wet and/or severe weather conditions, triggered by the major North Atlantic synoptic systems^19^. Besides, the climate of the area is strongly influenced by the Tyrrhenian Sea and by the complex orography of the Italian Peninsula.

Our aim is to:

- test for the occurrence of fundamental periodicities in the Gaeta pollen record, through REDFIT and wavelet transform analyses;
- characterise spatio-temporal patterns of vegetation dynamics, including the phasing of millennial-scale variability between the central and western Mediterranean;
- evaluate the role of past modes of climate variability in determining millennial-scale vegetation changes in the Mediterranean region.

## Results

### Vegetation history

**Figure 2:**
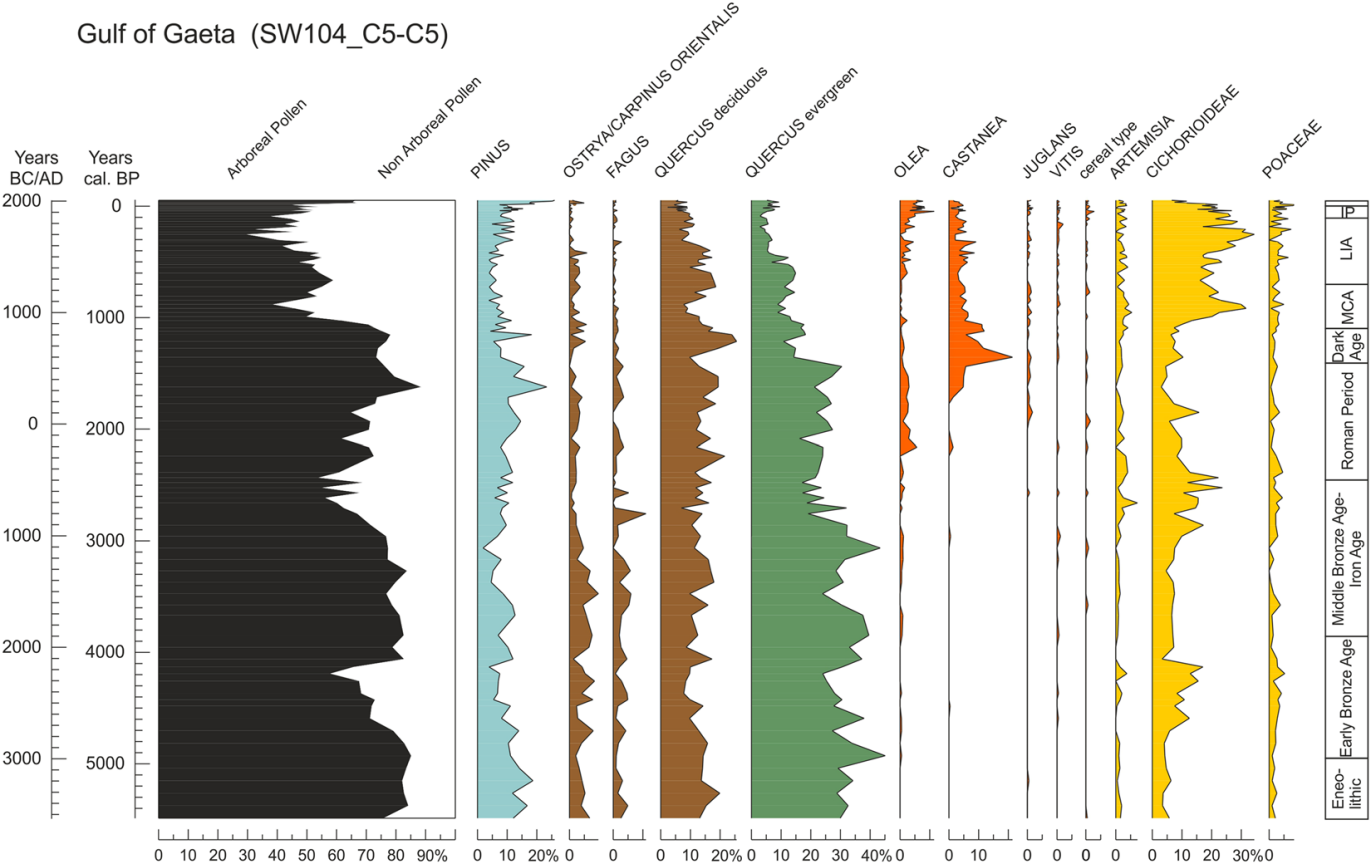
Pollen diagram of selected taxa from the Gaeta of Gaeta record (central Tyrrhenian Sea).

Over the last 5500 years, the vegetation history along the Gulf of Gaeta (Fig. 2) was characterized by repeated changes in the forest cover, vegetation structure and floristic composition^6,20^, which can be summarized as follows:

- From 5500 to 4750 cal BP the forest cover was composed of mixed temperate and Mediterranean forests, dominated by deciduous and evergreen *Quercus*.
- From 4750 to 4100 cal BP, a forest decline, culminating at around 4200 cal BP, was mainly related to a decrease in evergreen *Quercus*.
- From 4100 and 2900 cal BP, a new forest development shows a remarkable increase in evergreen and deciduous elements (mainly *Quercus*, *Ostrya*/*Carpinus orientalis*, and *Fagus*).
- From 2900 to 2300 cal BP, the forest vegetation declined once more, as indicated by an abrupt decrease in arboreal pollen (AP), reaching their minimum percentages between 2800 and 2600 cal BP.
- From 2300 to 1150 cal BP the pollen diagram suggests a remarkable forest expansion, partly due to the development of natural tree populations, including *Pinus*, and partly enhanced by arboricultural taxa *Castanea*, *Olea*, *Vitis* and *Juglans*, especially during and after the Roman times.
- From 1150 to 150 cal BP, a general forest decline is recorded. This trend was not progressive and steady; AP percentages display two forest decreases, from 1150 to 800 BP and from 400 to 150 BP, with an intervening moderate forest vegetation development from 800 to 400 BP related to a new oak-dominated woodland expansion.
- In the last two centuries, a new arboreal vegetation expansion was mostly related to an increase in *Pinus*, *Castanea*, *Olea*, *Vitis* and *Juglans*, which, together with other anthropogenic pollen indicators, highlight extensive agricultural practices and a marked human impact on the territory. On the whole, *Pinus* does not seem over-represented in the Gaeta record, in contrast to most marine sequences, as it increases mostly during the last centuries in relation to historically documented plantations.

Human impact became increasingly important in the record of the Gulf of Gaeta during the last two thousand years, probably playing a major role in the progressive general opening of the forest (Fig. 2). It is worth noting that a reliable representation of cereal pollen in marine pollen records is complicated by its low dispersal, masking both the real amount of regional cereal cultivation and the extent of human activities. Nonetheless, the main forest fluctuations displayed in the AP record correspond to the main historical climate fluctuations (e.g. LIA) and NAO index oscillations^20^. This suggests that climate may have been a main factor pacing the centennial- to millennial-scale phases of forest development and decline^6^.

### REDFIT and wavelet transform analyses

**Figure 3:**
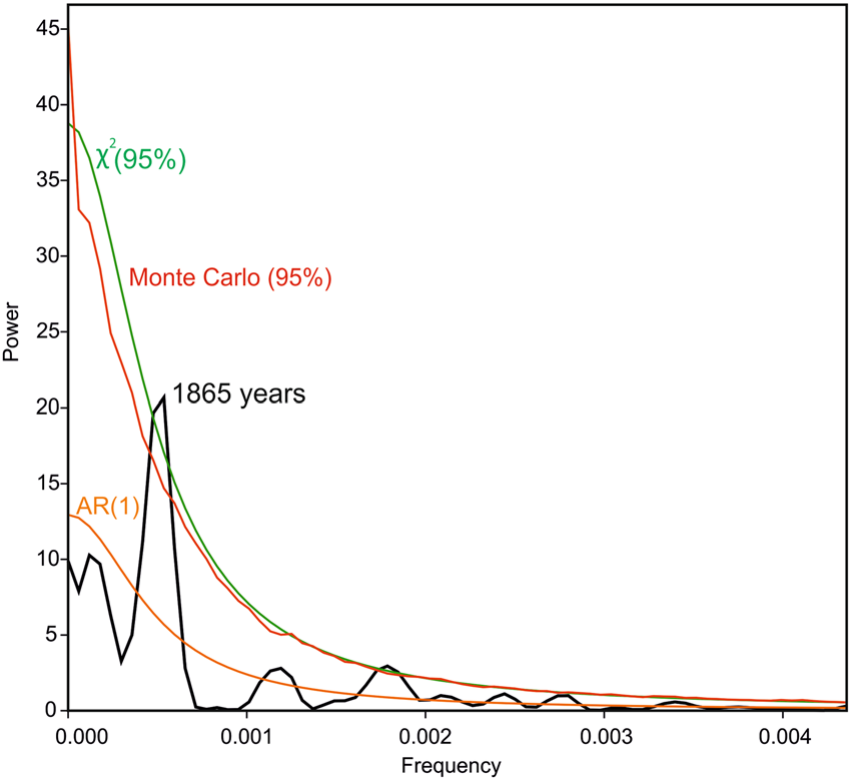
REDFIT spectral analysis of the Arboreal Pollen (AP) percentages of the Gulf of Gaeta record (black line). The time series is fitted to an AR^1^ red noise model (orange line). The 95% confidence levels of the χ2 and Monte Carlo tests are reported on the graph with a green line and a red line, respectively. The PAST 3.1 software program was used^64^.

The REDFIT spectral analysis applied to the Gaeta AP percentages exhibits a prominent peak in spectral power exceeding the 95% significance level of the confidence interval, corresponding to a periodicity of 1865 years (Fig. 3).

**Figure 4:**
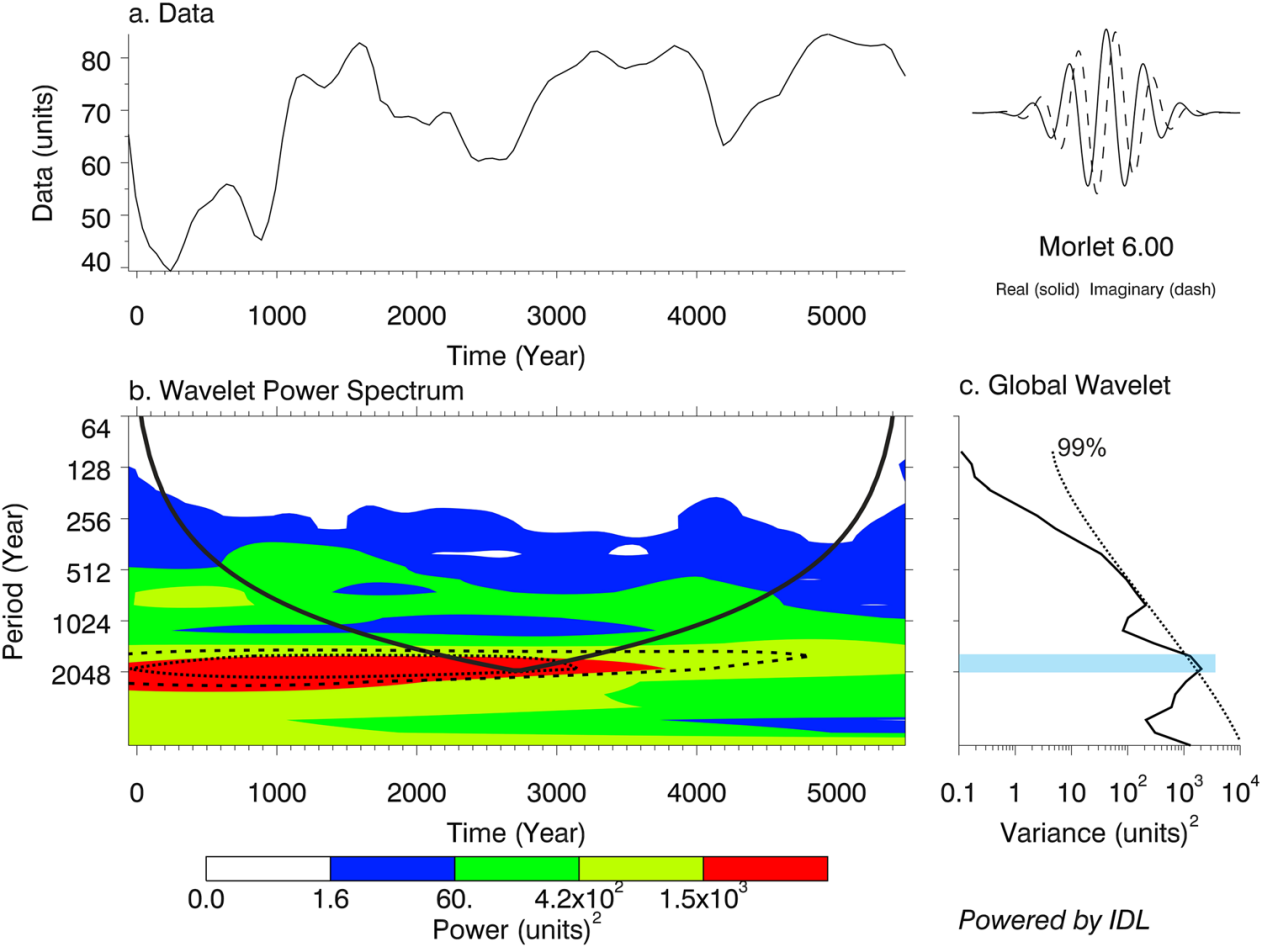
Wavelet transform of the AP percentages of the Gulf of Gaeta pollen record. The data were previously detrended and smoothed (**a**) using PAST 3.1 software program^64^. The wavelet transform was performed using the on-line interactive wavelet plot of the University of Colorado (http://paos.colorado.edu/research/wavelets/). The dashed lines shown by the Wavelet Power Spectrum (**b**) and Global Wavelet (**c**) graphs mark the 99% significance level of the variance. The black solid line in the Wavelet Power Spectrum (**b**) indicates the cone of influence.

The Wavelet transform analysis applied to the detrended AP time series of the last 5500 years highlights the dominance of a low frequency millennial oscillation (Fig. 4). The portion of frequencies exceeding the 99% significance level of the variance shows a bandwidth ranging from ca 2050 to 1670 years (ca. 1860 ± 190 years), consistent with the 1865-years periodicity peak obtained from the REDFIT analysis. Due to the low frequency of this periodic component relative to the length of the series, only a small part of the significant area falls inside the “cone of influence”. This means that the edge-padding with zero values may reduce the potential for detection of periodic components and suggests caution in the interpretation of the wavelet result. However, in light of the single dominant periodicity and excellent agreement with the REDFIT analysis, we consider the result to be robust.

## Discussion

### Periodicity

The millennial scale periodicity centred at ca. 1860-yr, detected by the statistical analyses of the Gaeta AP frequencies, is consistent with the ca. 1800-yr cycle recognized in several palaeoclimate and palaeoenvironmental records around the world (e.g.^7,21–23^). An 1823-yr cycle, originating from extreme oceanic tides associated with orbital coincidences, was held responsible for cooling at the sea surface by increasing vertical mixing in the oceans, so modulating global climate^24^. It would influence both the Ice-Rafted Debris (IRD) events, whose spectral analysis shows a high-power density in a broad band centred at about 1800 years^1^, and the climate events corresponding to the collapse of the Akkadian civilization (4.2 ka cal BP) and the LIA^24^. However, the global climatic implications of the tidal forcing have not been widely accepted^25^.

**Figure 5:**
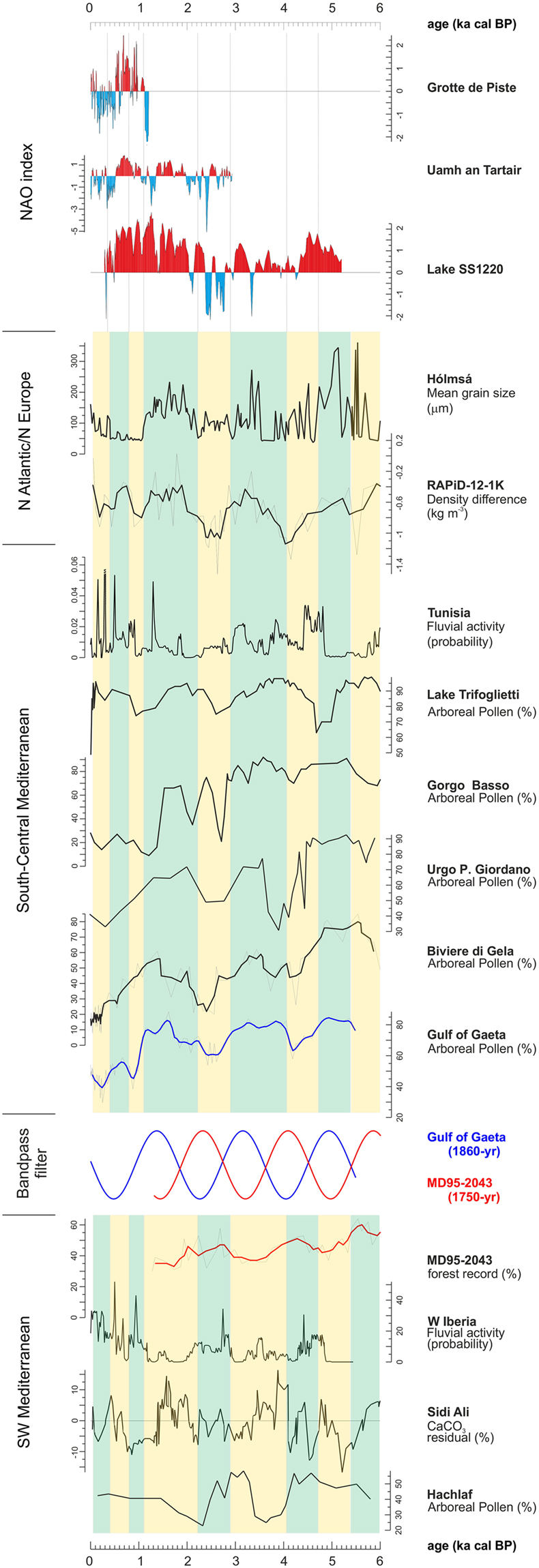
Palaeoclimate proxy records from the south-western Mediterranean, south-central Mediterranean, North Atlantic/Northern Europe, and NAO index. Hachlaf: Arboreal Pollen record^28^; Sidi Ali: detrended values [3^rd^ order polynomial] of carbonate data^23^; W Iberia: cumulative probability density plots of radiocarbon dates from floods and extreme fluvial event units^27^; MD95-2043: temperate and Mediterranean forest record, with three-point running mean in red^9^; Bandpass filter applied to the MD95-2043 (1750-yr filter) and Gulf of Gaeta (1860-yr filter) pollen records; Gulf of Gaeta (SW104_C5-C5): Arboreal Pollen record, with three-point running mean in blue^6,20^; Biviere di Gela: Arboreal Pollen record, with three-point running mean in bold^35^; Urgo Pietra Giordano: Arboreal Pollen record^36^; Gorgo Basso: Arboreal Pollen record^34^; Lake Trifoglietti: Arboreal Pollen record^37^; Tunisia: cumulative probability density plots of radiocarbon dates from floods and extreme fluvial event units^27^; core RAPiD-12-1K: upper ocean density stratification proxy^43^, with three-point running mean in bold; Hólmsá (Iceland): loess grain size record^41^; NAO index calculated from Lake SS1220, Greenland^11^; NAO index calculated from Uamh an Tartair, NW Scotland^32^; NAO index calculated from Grotte de Piste, Morocco^33^. Yellow bands correspond to generally arid time intervals; blue bands indicate wet periods.

Other authors have found a quasi-periodic ~1800-yr cycle. Debret *et al*.^7^ found statistically strong periodicities centred at 1700 and 1800 years, accompanied by periodicities of 1400 and 1500 years, in several paleoclimate records from the North Atlantic region during the last 6000 years, following the establishment of a cyclical internal oceanic forcing influenced by the Thermohaline Circulation (THC). Soon *et al*.^22^, through a cross-wavelet analysis applied to three main solar activity proxy time series (nitrate concentration, ^10^Be and ^14^C) show a strongly modulated and time-dependent signal for a ~1800-yr climate cycle (1885 years), together with a climate cycle of 1500-yr. The authors suggest that these cyclicities may correspond to fundamental solar modes influencing the global climate and producing an internal threshold response of the global THC to solar forcing. In the Mediterranean Basin, a direct counterpart of the periodicity found in the Gaeta pollen record is represented by the 1750-yr cycle (1740 ± 80 years) found by Fletcher *et al*.^9^ in recurrent episodes of forest declines in the MD95-2043 core from the Alboran Sea during the last 6000 years (Fig. 5).

The similarity of the periodicity found by Debret *et al*.^7^ and Fletcher *et al*.^9^ with the Gaeta record suggest the existence of common forcing influencing climate variability in the North Atlantic and western to central Mediterranean areas.

### Opposite spatio-temporal patterns in western and central Mediterranean

The MD95-2043^9^ and Gaeta pollen records show opposite patterns in the vegetation development/decline during the last millennia, suggesting that a common climate forcing was expressed differently in different sectors of the Mediterranean (Fig. 5).

The forest declines in the MD95-2043 record from the Alboran Sea have been explained with a NAO-related mechanism influenced by internal oscillations in interglacial Atlantic Meridional Overturning Circulation (AMOC) strength^9^. This may have determined changes of intensity and direction of the zonal winds represented by westerlies and the related hydroclimatic cycle of the western Mediterranean^9^, where a synchronism of Atlantic cooling phases (Bond events) and winter rain maxima was found at sub-millennial to centennial timescales with negative NAO-like conditions (^23^ and references therein). Similar oscillations recorded in the North African dust inputs, measured from the Alboran Sea core ODP site 976, confirm that the south-western Mediterranean region experienced intense centennial-scale changes in zonal winds^26^. This pattern is also reflected in the hydrological dynamics found in the CaCO_3_ record from Lake Sidi Ali in Morocco^23^ and in the curve of floods and fluvial activity in western Iberia^27^ (Fig. 5). Recurrent hydrological changes can be also inferred by the arboreal pollen record from Hachlaf in the Middle Atlas^28^, which shows a good correspondence with the changes in forest cover of the MD95-2043 record (Fig. 5). In continental Iberia, a remarkable resilience of the pine-dominated vegetation to millennial scale climate fluctuations may have partly masked this pattern in pollen records^29^. Consistent with present day NAO impacts described by Marshall *et al*.^30^, the Mediterranean northern borderlands experienced wet climate during phases of negative NAO-like conditions, as demonstrated in central-northern Italy for example by high lake levels from Lago dell’Accesa and phases of glacier advancements in the Apennines^31^.

Conversely, at Gaeta the main forest decline events, suggesting phases of decreased precipitation, correspond to phases of negative or declining positive values of NAO index^20^, as it appears from the three curves of NAO index reconstruction represented in Fig. 5^11,32,33^. Other pollen records from the south-central Mediterranean confirm the Gaeta pattern. The Sicilian sites of Gorgo Basso^34^, Biviere di Gela^35^, and Urgo Pietra Giordano^36^ show a remarkable consistency with the forest openings found at Gaeta at 4750–4100, 2900-2300, 1150-800, and 400-150 cal BP (Fig. 5). Although in the original publications these phases of forest decline were mostly interpreted as effects of human activity, the regional synchronicity of the vegetation dynamics points to a concomitant, or even predominant, influence of climate. The pollen record from Trifoglietti in S Italy^37^ precisely matches the openings at 2900-2300, 1150-800, and 400-150 cal BP, while the forest decline at 4750-4100 cal BP appears slightly earlier, being found at 5200-4500 cal BP (Fig. 5). At Lago Grande di Monticchio, the 4.2 ka event is not clearly visible, but a slight decline occurs at 2900-2400 cal BP and a reduction in forest is found at 1100 cal  BP^38^. By contrast, the 4700-4100 cal BP event is clearly visible in tree records from the marine core MD04-2797CQ from the Siculo-Tunisian Strait^4^, and from the coastal sites of Lago Alimini Piccolo^10^ and Lago Battaglia^39^. Besides, the decreases in floods and fluvial activity from Tunisia^27^ generally match the forest openings in the pollen records from the south-central Mediterranean (Fig. 5).

The late Holocene vegetation changes recorded in Mediterranean sites are mostly explained by human activity, which is taken for granted since agro-pastoral and silvicultural practices, forest clearance, fires and human settlements are documented up to present time. For example, a dramatic lowering of lake level at Lake Preola in Sicily^40^, coeval to a decline in forest vegetation also recorded in the other Sicilian sites, was interpreted as a possible effect of human activity due to overexploitation of water resources for cultivation^36^, despite the contemporary occurrence of the well-known 4.2 ka “Bond 3” climate event. Our data suggest that near-coeval forest fluctuations, recorded over wide Mediterranean regions, were cadenced by well-known climate fluctuations, identified also by independent climate proxies at a global scale, thus emphasizing the role of climate on the vegetational landscape.

**Figure 6:**
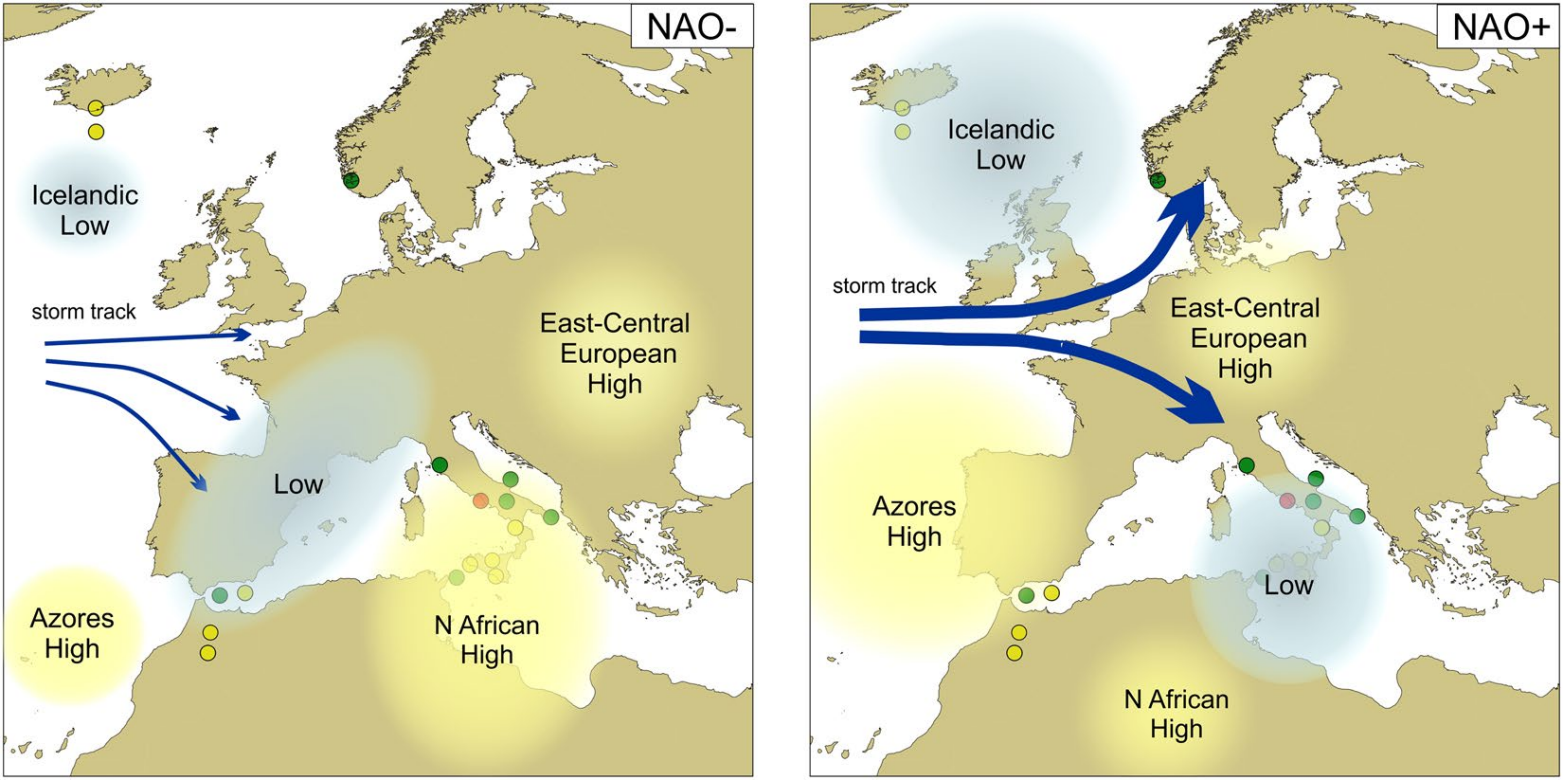
Tentative reconstruction of atmospheric configurations over Europe and North Africa in correspondence with negative (left) and positive (right) NAO index. Blue arrows indicate the direction and intensity of storm tracks. Possible positions of the Azores High, North African High, Central European High, and Icelandic Low are also indicated, together with low pressure areas with precipitations. The Gaeta record is represented by a red dot, sites shown in Fig. 5 by yellow dots and other sites mentioned in the text by green dots. The background map (Mollweide projection) was retrieved from Natural Earth, a free vector and raster map data of public domain (http://www.naturalearthdata.com). The final map was generated using both Corel Draw Suite X8 (https://www.coreldraw.com) and QGIS version 2.18.15 ‘Las Palmas’ (QGIS Development Team, 2017. QGIS Geographic Information System. Open Source Geospatial Foundation Project https://www.qgis.org/it/site/).

The Gaeta record shows an excellent correspondence with proxy records of wind strength and precipitation at high North Atlantic latitudes (Fig. 5). During intervals of prevailing strong westerly flow, a northward shift of Atlantic storm tracks resulted in both greater wind intensities over Iceland (grain size record from the Hólmsá loess profile in southern Iceland^41^) and higher winter precipitation in Norway (past glacier fluctuations in northern Folgefonna^42^). These millennial-scale fluctuations also match the upper ocean density stratification in the subpolar North Atlantic core RAPiD-12-1K, where increased (decreased) stratification occurred during intervals of inferred weak (strong) zonal flow^43^ (Fig. 5). In support of this climatic teleconnection, besides the Gaeta record, we found a statistically significant ~1800-year cycle also in the south-central Mediterranean pollen record from Biviere di Gela^35^ and in the density difference record from North Atlantic core RAPiD-12-1K^43^, whose REDFIT analyses are reported in Supplementary Fig. S1. Summarizing, during intervals of positive NAO index there was increased moisture both in the south-central Mediterranean and at high North Atlantic latitudes, as indicated by forest expansions and other climate proxies. At the same time, dry conditions, determining openings of forests, are recorded in south-western and in north-central Mediterranean (Fig. 6).

The precipitation patterns found in the Mediterranean may be explained in different ways, involving the Atlantic climate system and/or the subtropical atmospheric circulation, acting in different seasons.

### Atlantic climate processes

The alternation of forest increases and declines in the south-central Mediterranean may represent the effect of periodic variations and latitudinal/longitudinal displacement of the North Atlantic pressure centers and the westerlies, with consequent changeable penetration of storm tracks into the Mediterranean region, in relation to the prevailing or predominant phases of NAO-like circulation (Fig. 6).

A weakened westerly flow (NAO negative), resulting in a convergence of winter Atlantic storm tracks into central-western Europe may have promoted increased winter precipitation and forest development in the western Mediterranean (Fig. 6). At the same time, a weakened westerly flow may have led to a reduction of the Icelandic winds and precipitation over western Norway and of forest development and precipitation in the south-central Mediterranean. Although a correlation of a negative NAO index with decreased humidity in the south-central Mediterranean is not always predicted by recent climatological data^15^, the consistency of the Gaeta pollen record with the NAO index curves and proxies is a valuable hint in support of this hypothesis for the past. Based on modern data, a positive correlation between NAO and precipitation was documented by Brandimarte *et al*.^17^ in eastern Sicily, ca. 3 degrees south of the Gulf of Gaeta. In the past, this positive correlation may have influenced more northerly regions, as latitudinal fluctuations of contrasting hydrological sectors may be expected in response to changing NAO^5^ and the spatial centres of climate impact may vary over time.

Another possible explanation for the aridity phases in the south-central Mediterranean involves the extension and location of the North African anticyclone, often neglected in palaeoclimatic models, but well-known to meteorologists for the torrid temperatures and dryness it may induce especially in summer (cf. Spring/Summer 2012 and 2017). At 4.2 ka cal BP, a weak NAO circulation (NAO negative) appears to correspond to a prolonged position of the North African anticyclone on the south-central Mediterranean^10,20^, which in turn may have represented an important atmospheric blocking for the westerly storm track (Fig. 6). This interaction between NAO circulation and North African anticyclone dynamics may have enhanced the climate contrast between the south-central Mediterranean and the regions in northern-central and western Mediterranean. Similar atmospheric configurations possibly occurred also during other negative NAO phase intervals, such as the 2.8 ka cal BP and the LIA. The establishment of atmospheric blockings in North Atlantic and North Africa during negative NAO phases, highlighted by the model of Shabbar *et al*.^44^, seems to support this atmospheric configuration.

An interaction between NAO phases and North African high pressures is reflected in Saharan dust records of Mediterranean Northwest Africa: Zielhofer *et al*.^45^ show a strong relationship between late Holocene dust peaks, positive NAO, and Saharan air circulation, concomitant with western Mediterranean arid phases. A combined activity of NAO circulation and extension/migration of the North African anticyclone accounting for the aridity phases in the western Mediterranean, may explain the observed opposite climate pattern.

The structure and climate impact of the NAO may be also significantly influenced by the East Atlantic (EA) pattern, which can modulate the precipitation over Europe and play a role in the positioning of the primary North Atlantic storm track and jet streams^46,47^. After the NAO, the EA represents the dominant mode of winter precipitation variability in the Mediterranean region, and, unlike the NAO, the EA accounts for opposed precipitation patterns between the west and south-central Mediterranean sectors^48^. The interplay of NAO and EA with the same phase may account for similar precipitation patterns between Northern Europe and south-central Mediterranean and opposite precipitation patterns between south-central Mediterranean and western Mediterranean^49^, as those recorded in Fig. 5. NAO-EA configuration phases may have been influenced also by solar activity, since solar irradiance was suggested to determine the sign of the EA phase, with negative (positive) phases associated with low (high) solar irradiance, as probably occurred during LIA (and MCA)^49^. Thus, the decreases in precipitation recorded in central Mediterranean, most of which correspond to solar grand minima^50^ and negative NAO index (Fig. 5), may represent phases of both negative NAO and EA, so supporting the different precipitation spatial patterns.

A different distribution of the local cyclogenesis may also account for this pattern. There are two types of cyclogenesis in the Mediterranean: one acting in Winter, which is strongly dependent on North Atlantic synoptic activity influenced by NAO, and the other mostly acting in summer, with local precipitation cells developed in relation to air-sea temperature contrasts^19^. The Gulf of Genoa, in the central Mediterranean, represents by far the most active area in the whole Mediterranean Basin in winter, between November and February. In contrast, one of the most active areas of Summer cyclogenesis is located in the Iberian Peninsula. This opposite cyclogenesis seasonality may have determined different responses of vegetation in the western and central Mediterranean in the past and may represent a topic for future palaeoclimate research.

### Subtropical and global climate processes

Climate factors influencing the tropical and subtropical regions may have also played a role in determining the observed climatic pattern, especially the ITCZ latitudinal shifts.

Strong covariation between ITCZ position and NAO records reveals a tight coupling between these two synoptic weather and climate phenomena over decadal-to-centennial timescales^51^. Southward migrations of the ITCZ occur when the North Atlantic region is relatively cold, during Bond events corresponding to negative NAO index, owing to enhanced high-latitude ice cover and a slowdown of the AMOC^51,52^. In correspondence with the Atlantic cooling phases, for example during the 4.2 ka event and the LIA, corresponding to Bond events 3 and 0, respectively, the ITCZ migrated southward determining planetary climate changes^53–55^, revealed also by the deforestation events in the Gaeta pollen record (Fig. 5).

In addition, the recurrence of a ca. 1800-year cycle in many records around the world^22^ points to global climate factors as possible forcing for the observed precipitation pattern of the Mediterranean. Two main candidates may be the external forcing by solar activity changes and the internal long-term ocean/atmosphere feedbacks of the THC/AMOC.

Solar activity, which shows inherent periodic changes at millennial to decadal time scales, is able to modulate both NAO and the position of the ITCZ^56^. In the central Mediterranean, these cycles influence both hydrological and environmental conditions^57^, which in turn affect vegetation^58,59^. At a millennial scale, fundamental solar cycles are found at 2300-yr (Hallstattzeit cycle) and 1000-yr (Eddy cycle). However, Soon *et al*.^22^ suggest the possibility that also a ca. 1800-yr cycle may be related to solar forcing, possibly representing a fundamental cycle connected to intrinsic variations in solar radiative and charged particle output.

Soon *et al*.^22^ also speculate that the 1800-yr cycle may be of derived nature, representing an internal threshold response of the THC/AMOC circulation to external solar forcing, able to promote significant climate fluctuations around the globe. A recent model documents positive feedbacks between the AMOC and the NAO, which shows a coupled variability and quasi-synchronous interactions^60^. In the central Mediterranean Basin, the influence of AMOC is evoked to explain climatically-driven environmental changes related to the advection of moisture, possibly resulting from variability in the strength and latitudinal trajectory of the westerlies, most of which chronologically consistent with forest declines in the south-central Mediterranean pollen sites considered in this work^31^.

Although much work is still required to characterize fully the nature of these intricate climate interactions, growing evidence suggests that the periodicity of ca. 1800-yr found in Gaeta (Figs 3–5) and in other Mediterranean records (Figs 5 and S1) may be a prominent expression of the global climate.

## Conclusion

The new detailed and chronologically well-constrained pollen record from the Gulf of Gaeta, in the Tyrrhenian Sea, has revealed a recurrent pattern of forest dynamics with a cyclicity of approx. 1860 years. The vegetation development at Gaeta is consistent with other pollen records from the south-central Mediterranean, a region especially sensitive to climate change, being under the influence of both the North Atlantic circulation and the high-pressure system of North Africa. In fact, some of the forest declines previously attributed to anthropogenic impact may be linked to this slow-changing component of moisture availability for plant growth. At the same time, the Gaeta record shows a striking antiphase correspondence with the pollen record from core MD95-2043 in the Alboran Sea, in south-western Mediterranean. This contrasting pattern is confirmed by other climate proxy records from the south-central and south-western Mediterranean, respectively, including carbonate in sediments and fluvial activity, which show alternate wet and dry phases during the last 6000 years. At much higher latitudes, in Iceland and Norway, a succession of environmental changes and periodicity similar to the Gaeta record have also been observed.

The correspondence in the series of events of such different records suggests that the explanation of the recurrent palaeoenvironmental changes at Gaeta may have implications well beyond site-specific interests. Thus, we have reached the following conclusions:

- Periodicity: the Gaeta record contributes to a growing body of evidence supporting the existence of a ca 1800 yr climate fluctuation during the mid- to late Holocene. Although it is necessary to document in different paleoenvironmental frameworks the nature of this climate periodicity, the convergence of many different sources of evidence towards a 1800-yr cycle strongly suggests that the recurrent vegetation changes in the Gaeta record may have been induced by large-scale changes in climate modes, linked either to changes in solar activity and/or AMOC intensity, influencing the water availability needed for forest expansions.
- Spatio-temporal pattern: the evidence for millennial scale variability in the Gaeta vegetation encompasses the late Holocene, despite a widespread human activity on the territory, which induced a general decline in the forest cover without completely obliterating recurrent vegetation dynamics driven by natural factors at a regional scale. Although the human impact has exerted an ever-increasing pressure on the natural landscape, the fluctuations in the forest vegetation appear strongly cadenced by climate changes identified also in other proxy records. The same patterns are detected not only in marine pollen records, but also in lacustrine sites, and in other palaeohydrological independent proxy-records from the south-central Mediterranean, latitudinally ranging from Tunisia to southern Italy. These records clearly show dry intervals in correspondence with specific well-known climate events, including the 4.2 ka event, the Medieval Climate Anomaly and the Little Ice Age, but also highlight the relevance of other climate spells, often neglected in the literature including, for example, a deforestation coeval to the so-called “Bond 2” event around 2.8 ka cal BP. The clearly opposed trends observed in several palaeohydrological records from the south-western Mediterranean, indicating generally wet climate conditions during the dry spells found in the Gaeta record, suggest that different expressions of climate modes occurred in the south-western and south-central Mediterranean at the same time. Complex spatial patterns of atmospheric circulation may have acted over the Mediterranean regions.
- Climate processes: a clear temporal correspondence between phases with negative (positive) NAO index and forest declines (increases) in the Gaeta pollen record indicates that the prevailing or predominant phases of NAO-like circulation were prominent factors inducing hydrological variations in the south-central Mediterranean, through changes in zonal winds and different storm track penetration. However, the observed contrasting hydrologic regimes and vegetation dynamics point also to more complex configuration of the atmospheric circulation, including the EA pattern and its interplay with the NAO, as well as the North African anticyclone dynamics. At a larger geographical scale, considering the tropical engine, displacement of the ITCZ may also have indirectly influenced the recurrent changes observed in the palaeoenvironmental records.

The new palaeoenvironmental data from the south-central Mediterranean confirms a higher latitudinal and longitudinal complexity of atmospheric circulation patterns than generally supposed, and an intricate interaction of several forcing factors. A consequence of this complexity is that it may not be appropriate to include *tout-court* “the Mediterranean” in the models of past climate variability, because, in the past as at present, the expression of atmospheric patterns was different in the south-western and south-central Mediterranean.

Part of the challenge for the future research on millennial scale climate variability of the Mediterranean is unravelling the seasonal components and linkages between temperate (wintertime) systems and the tropical (summertime) systems. Although different parts of the Mediterranean show contrasting moisture signals, they may also be reflecting subtle differences in the seasonal timing of precipitation generation, which new models will have to be able to predict, also in the light of the strong seasonal feature of the Mediterranean cyclogenesis centers.

## Materials and Methods

### Sampling

The record from the Gulf of Gaeta is composed of the two cores SW104_C5 (40°58′24.993″N, 13°47′03.040″E; 108 cm long) and C5 (40°58′24.953″N, 13°47′02.514″E; 710 cm long), recovered at a distance of 15 km from the Campanian coast (central Tyrrhenian Sea) at 93 m below sea level and ca. 10 m from each other, during the AMICA2013 oceanographic cruise onboard the R/V Urania of CNR^6^ (Figs 1 and 2). The stratigraphic correlation of the two cores was based on magnetic susceptibility signals and was facilitated by the recognition of a distinct common peak in magnetic susceptibility of the two cores, found at 61 and 48 cm depth in SW104_C5 core and C5 core, respectively, corresponding to the tephra layer of the Vesuvius eruption dated at 1906^6^.

### Chronology

The chronology of the sedimentary record is mostly based on ^210^Pb and ^137^Cs radionuclides measurements for the uppermost 60 cm and the identification, supported by geochemical analyses, of five tephra layers at the depths of 53, 319, 403, 414 and 437 cm, namely: Vesuvius (1906 AD), Vateliero-Ischia (2.4–2.6 ka BP), Capo Miseno (3.7–3.9 ka BP), Astroni3 (4.1–4.3 ka BP), and Agnano M. Spina (4.42 ka BP)^6^. Radiocarbon dating is not available because of low carbon content in the sediments. The age-depth model takes also into account reliable time-constrained biostratigraphic events specific to the Tyrhenian Sea, such as the peak in abundance of *Globorotalia truncatulinoides* left coiled (1718 ± 10 yr AD) and the acme interval of *Globigerinoides quadrilobatus* (base 3.7 ± 0.048 ka BP, top 2.7 ± 0.048 ka BP). Other chronological tie points to set the age-model were also derived from the comparison of the δ^18^O_*G.ruber*_ of our cores and the C90 core from the close Gulf of Salerno. The good visual correlation between the δ^18^O_*G.ruber*_ signal from our site with data from the southern Tyrrhenian Sea, Gulf of Taranto, Adriatic Sea, and the eastern Mediterranean strongly support the robustness of the proposed age-model^6,20^.

### Pollen analysis

Pollen analysis was carried out on the uppermost 512 cm of the composite sequence, mostly composed of fine-grained light grey hemipelagic sediments. A total of 100 samples were chemically treated with HCl (37%), HF (40%) and NaOH (20%). The main percentage sum is based on terrestrial pollen excluding aquatics and non-pollen palynomorphs. Excluding pollen of aquatics, fern spores and other non-pollen palynomorphs (NPPs), an average number of ca. 200 pollen grains of terrestrial plants per sample was counted. Pollen assemblage zones were visually determined. The computer program Psimpoll 4.27^61^ was used to plot the pollen diagram.

### Statistical analyses

A REDFIT spectral analysis^62^ and a Wavelet transform^63^ were applied to time series based on the Arboreal Pollen percentage values from the Gaeta record, in order to detect the possible occurrence of a fundamental tempo in the forest cover extent variability. REDFIT allows direct processing of unevenly spaced time series and, hence, the usual prerequisite of data interpolation is not required. The REDFIT spectral analysis was carried out on the raw percentages of the Arboreal Pollen (AP) using PAST 3.13 software^64^, through a Welch method with an oversampling factor of 3 and one data segment. The time series was fitted to an AR^1^ red noise stochastic model, and the significance of the frequency peak was tested using both a χ^2^ test and a Monte Carlo simulation.

The Wavelet transform is a technique used for the identification of spectral signatures in palaeoclimate time series, with the particular advantage of describing non-stationarities, i.e. discontinuities and changes in frequency or magnitude through time^63^. This procedure requires continuous data with even spacing of points. For this reason, prior to wavelet analysis the AP percentages of the Gaeta record were detrended using a smoothing spline method with PAST 3.1. The new time series was obtained by interpolating the smoothed AP record every 50 years, a time interval representing the mean time resolution of the original AP record. This does not increase the number of degrees of freedom that make the cycles more statistically significant than they actually are, so avoiding temporal autocorrelation in the data. The Morlet method was chosen for the continuous wavelet transform. The data series was zero-padded in order to avoid boundary effects and spectral leakage produced by the finite length of the time series.

### Data availability

The data from Hachlaf^29^; Sidi Ali^23^; W Iberia floods and extreme fluvial events^27^; MD95-2043: temperate and Mediterranean forest record^9^; Biviere di Gela^35^; Urgo Pietra Giordano^36^; Gorgo Basso^34^; Lake Trifoglietti^37^; Tunisia: floods and extreme fluvial events^27^; core RAPiD-12-1K upper ocean density stratification proxy^43^; Hólmsá (Iceland) loess grain size record^41^; NAO index from Lake SS1220, Greenland^11^; NAO index from Uamh an Tartair, NW Scotland^32^, and NAO index from Grotte de Piste, Morocco^33^ were recovered from the original publications.

The pollen data from the Gulf of Gaeta (SW104_C5-C5)^6,20^ are available from the corresponding author on reasonable request.

## Electronic supplementary material

Supplementary Information
