# Supplementary material for: Holocene forest dynamics in central and western Mediterranean: periodicity, spatio-temporal patterns and climate influence
Source: Sci Rep. 2018 Jun 12;8:8929. doi: 10.1038/s41598-018-27056-2 (PMC5997640; doi:10.1038/s41598-018-27056-2)
Supplement: Supplementary file 1 — Supplementary Information [file 41598_2018_27056_MOESM1_ESM.pdf]

Supplementary information

Holocene forest dynamics in central and western Mediterranean: periodicity, spatio-temporal patterns and climate influence

Federico Di Rita [a*], William J. Fletcher [b], Josu Aranbarri [c], Giulia Margaritelli [d], Fabrizio Lirer [d], Donatella Magri [a]

[a] Department of Environmental Biology, Sapienza University of Rome, Piazzale Aldo Moro, 5, 00185, Roma, Italy

[b] Department of Geography, School of Environment, Education and Development, The University of Manchester, Oxford Road, Manchester, M13 9PL, UK

[c] Department of Geography, Prehistory and Archaeology, University of Basque Country, C/ Tomás y Valiente s/n, 01006 Vitoria-Gasteiz, Spain

[d] Istituto per l'Ambiente Marino Costiero (IAMC), Consiglio Nazionale delle Ricerche, Calata Porta di Massa, Interno Porto di Napoli, 80133 Napoli, Italy

* Corresponding author:

Federico Di Rita
Department of Environmental Biology
Sapienza University of Rome
Piazzale Aldo Moro, 5
00185, Roma, Italy
e-mail: federico.dirita@uniroma1.it

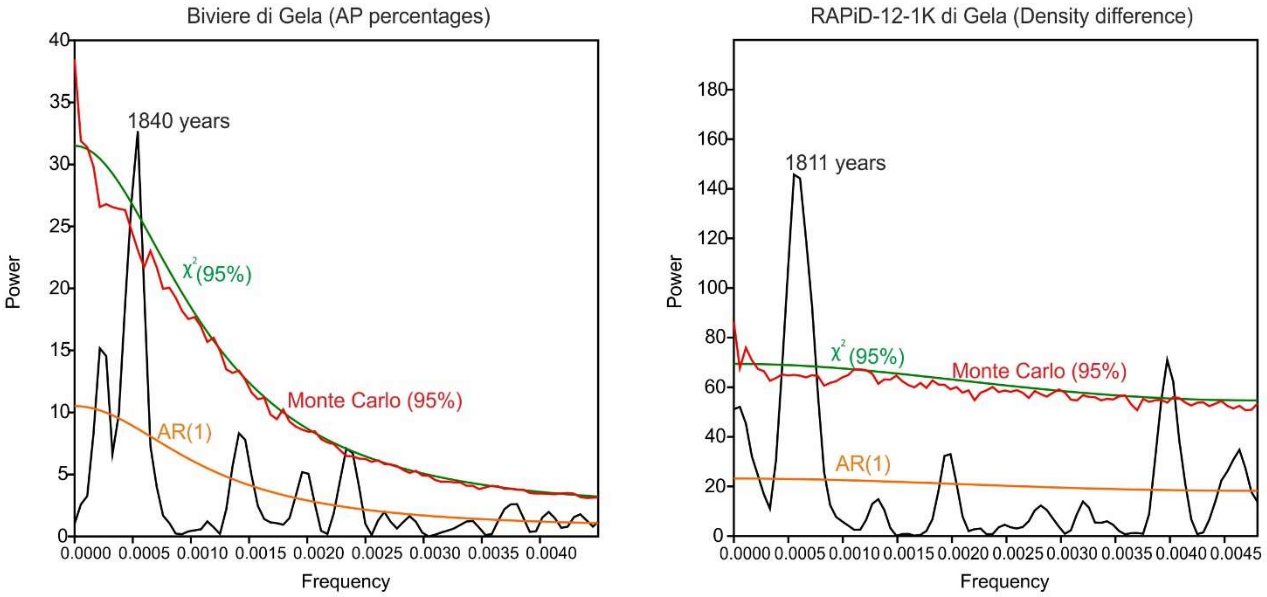

Figure S1. **REDFIT spectral analysis of Holocene palaeoclimate proxy records shown in Fig. 1.** (A) Biviere di Gela pollen record: AP percentages (35). (B) core RAPiD-12-1K: density difference (kg m^{-3}) upper ocean density stratification proxy (43). A Welch method with an oversampling factor of 3 and one data segment. The time series is fitted to an AR (1) red noise model (orange line). The 95% confidence levels of the χ2 and Monte Carlo tests are reported on the graph with a green line and a red line, respectively. The PAST 3.1 software program was used (64).
